# Supplementary material for: The Systems Biology Research Tool: evolvable open-source software
Source: BMC Syst Biol. 2008 Jun 29;2:55. doi: 10.1186/1752-0509-2-55 (PMC2446383; doi:10.1186/1752-0509-2-55)
Supplement: Additional file 1 — SBRT Archive. An archive of the current version of the Systems Biology Research Tool. [file 1752-0509-2-55-S1.zip › sbrt-1.4.0/doc/developers_guide/api/sbrt/kernel/fba/SinkRxn.html]

SinkRxn


|  |  |  |
| --- | --- | --- |
| **Overview** \| **Package** \| **Class** \| **Use** \| **Tree** \| **Deprecated** \| **Index** \| **Help** | |  |
| **PREV CLASS**   **NEXT CLASS** | **FRAMES**    **NO FRAMES**     **All Classes** |  |
| SUMMARY: NESTED \| FIELD \| CONSTR \| METHOD | DETAIL: FIELD \| CONSTR \| METHOD |  |


---


## sbrt.kernel.fba Class SinkRxn

```
java.lang.Object
  sbrt.kernel.fba.ExchangeRxn
      sbrt.kernel.fba.SinkRxn
```

**All Implemented Interfaces:**: ChemRxn

---

``` public final class SinkRxn extends ExchangeRxn ```

This class is used to represent sink reactions in Flux Balance Analysis. A
sink reaction is a type of exchange reaction that consumes a particular
chemical species, but does not supply it.

Instances of this class are immutable.

**Author:**
:   This class was written and documented by Jeremiah Wright while in the
    Wagner lab.

---

**Constructor Summary**

`SinkRxn(java.lang.String chemSpecies)`
  
          Constructs a new sink reaction for the provided chemical species.


| **Method Summary** | |
| --- | --- |
| `LinearComb<java.lang.String>` | `getReactants()`             Returns a single-term linear combination. |
| `boolean` | `isEquivalent(ChemRxn rxn)`             Indicates if the provided chemical reaction is stoichiometrically equivalent to this sink reaction. |
| `boolean` | `isReverse(ChemRxn rxn)`             Indicates if the provided chemical reaction is the reverse of this sink reaction. |
| `java.lang.String` | `toString()`             Returns a string representation of this sink reaction. |

**Methods inherited from class sbrt.kernel.fba.ExchangeRxn**

`equals, getProducts, getSpecies, hashCode`

**Methods inherited from class java.lang.Object**

`clone, finalize, getClass, notify, notifyAll, wait, wait, wait`

**Constructor Detail**

### SinkRxn

```
public SinkRxn(java.lang.String chemSpecies)
```

:   Constructs a new sink reaction for the provided chemical species.

    **Parameters:**: `chemSpecies` - the chemical species this sink consumes.


**Method Detail**

### getReactants

```
public LinearComb<java.lang.String> getReactants()
```

:   Returns a single-term linear combination. The variable
    of this term is the consumed chemical species and the coefficient is 1.0.

    :   **Specified by:**: `getReactants` in interface `ChemRxn` **Overrides:**: `getReactants` in class `ExchangeRxn`
    :   **Returns:**: a single-term linear combination. The variable of this term is the consumed chemical species and the coefficient is 1.0.

---


### isEquivalent

```
public boolean isEquivalent(ChemRxn rxn)
```

:   Indicates if the provided chemical reaction is stoichiometrically
    equivalent to this sink reaction. Sinks can only be equivalent to other
    sinks (of type `SinkRxn`). Two sink rections are equivalent only
    if they consume the same chemical species.

    :   **Parameters:**: `rxn` - the chemical reaction to test for equivalence. **Returns:**: `true` if both reactions are equivalent; `false` otherwise.

---


### isReverse

```
public boolean isReverse(ChemRxn rxn)
```

:   Indicates if the provided chemical reaction is the reverse of this sink
    reaction. The only possible reverse reactions of sink reactions are
    source reactions (SourceRxn). A sink and source are the
    reverse of each other only if their associated chemical species is the
    same.

    :   **Parameters:**: `rxn` - the chemical reaction to test. **Returns:**: `true` if the provided reaction is the reverse of this reaction; `false` otherwise.

---


### toString

```
public java.lang.String toString()
```

:   Returns a string representation of this sink reaction.

    :   **Specified by:**: `toString` in interface `ChemRxn` **Overrides:**: `toString` in class `java.lang.Object`
    :   **Returns:**: a string representation of this sink reaction.


---


|  |  |  |
| --- | --- | --- |
| **Overview** \| **Package** \| **Class** \| **Use** \| **Tree** \| **Deprecated** \| **Index** \| **Help** | |  |
| **PREV CLASS**   **NEXT CLASS** | **FRAMES**    **NO FRAMES**     **All Classes** |  |
| SUMMARY: NESTED \| FIELD \| CONSTR \| METHOD | DETAIL: FIELD \| CONSTR \| METHOD |  |


---
